# Improvement of irradiation-induced fibroblast damage by α2-macroglobulin through alleviating mitochondrial dysfunction

**DOI:** 10.1080/13880209.2022.2096077

**Published:** 2022-07-26

**Authors:** Chaoji Huangfu, Nan Tang, Xiaokun Yang, Zhanwei Gong, Junzheng Li, Junting Jia, Jingang Zhang, Yan Huang, Yuyuan Ma

**Affiliations:** a Center for Disease Control and Prevention, Western Theater Command, Lanzhou, PR China; b School of Nursing, Lanzhou University, Lanzhou, PR China; c Department of Emergency Medicine, The General Hospital of Western Theater Command, Chengdu, PR China; d NMPA Key Laboratory for Quality Control of Blood Products, Institute of Health Service and Transfusion Medicine, Academy of Military Medical Sciences, Beijing, PR China; e Department of Neurology, Chengdu Third People’s Hospital, Chengdu, PR China

**Keywords:** TRPM2, plasma protein, plasma product, proteinase inhibitor

## Abstract

**Context:**

α2-Macroglobulin (α2-M) is believed to be a potential anti-irradiation agent, but related mechanisms remains unclear.

**Objective:**

We investigated the irradiation protective effect of α2-M.

**Materials and methods:**

A total of 10 Gy dose of irradiation was used to damage human skin fibroblasts. The influence of α2-M (100 µg/mL) on the proliferation, migration, invasion and apoptosis of fibroblasts was observed using Cell Counting Kit-8 (CCK8), wound healing, transwell, and flow cytometry. Malondialdehyde, superoxide dismutase and catalase was measured using related ELISA kits. The levels of mitochondrial membrane potential and calcium were detected using flow cytometry. The expression of transient receptor potential melastatin 2 (TRPM2) was investigated through western blotting and immunofluorescence staining.

**Results:**

High purity of α2-M was isolated from Cohn fraction IV. α2-M significantly increased cell proliferation, migration, invasion, but suppressed cell apoptosis after irradiation. The promotion of cell proliferation, migration and invasion by α2-M exceeded over 50% compared group irradiation. The increased cell ratio in the S phase and decreased cell ratio in the G2 phase induced by irradiation were remarkably reversed by α2-M. α2-M markedly suppressed the increased oxidative stress level caused by irradiation. The mitochondrial damage induced by irradiation was improved by α2-M through inhibiting mitochondrial membrane potential loss, calcium and TRPM2 expression.

**Discussion and conclusions:**

α2-M significantly promoted the decreased fibroblast viability and improved the mitochondria dysfunction caused by irradiation. α2-M might present anti-radiation effect through alleviating mitochondrial dysfunction caused by irradiation. This study could provide a novel understanding about the improvement of α2-M on irradiation-induced injury.

## Introduction

α2-Macroglobulin (α2-M) is a 720-kDa plasma protein, and it is synthesized mainly by hepatocytes. α2-M is known as a kind of proteinase inhibitor, and it has been believed to be involved in several pathological and physiological processes including coagulation system balance, anti-radiation, antitumor and inflammation (Huangfu et al. 2016b). Meanwhile, α2-M could bind to some cytokines, such as nerve growth factor (NGF), basic fibroblast growth factor (bFGF), platelet-derived growth factor (PDGF) and further affect their functions (Huangfu et al. 2016a).

Some studies have reported the regulatory roles of α2-M in irradiation-induced injury. It was reported that α2-M could protect human bone marrow mesenchymal stem cells from irradiation damage through inhibiting pluripotent differentiation injury, autophagy and promoting osteogenic differentiation (Liu et al. 2018). α2-M could alleviate bone exposure, alopecia, and inflammation in the jaw osteoradionecrosis rats (Li et al. 2019). These reports suggest that α2-M plays an important role in the anti-radiation progress. However, the specific mechanism remains unclear.

The wound healing of radiation ulcer is closely related to the proliferation and migration of fibroblasts, granulation tissue formation and collagen synthesis (Mao et al. 2016; Aschermann et al. 2017). Fibroblasts play an important role in the process of radiation wound repair through proliferation and migration. Fibroblasts could synthesize and secret a large number of collagen fibres, and facilitate the repair process of skin injury caused by irradiation (Hasegawa et al. 2019; Suto et al. 2019). However, if α2-M could influence the viability of fibroblasts and further improve irradiation-induced injury remain unclear.

Several studies have reported that irradiation could lead to the damage of mitochondria, which is closely related with cell apoptosis, Ca^2+^ homeostasis, redox regulation and adenosine triphosphate synthesis. Meanwhile, the damage of mitochondria DNA could influence the next generation (Wang et al. 2019). However, if α2-M could improve the function of mitochondria after irradiation has not been reported.

This study investigates the influence of α2-M on the cell proliferation, apoptosis of fibroblasts, reactive oxygen species (ROS) levels and mitochondrial function improvement after irradiation. Meanwhile, the protein expression of H2AX and TRPM2 was measured after irradiation and α2-M treatment. This research could further uncover the regulatory role of α2-M protecting against irradiation injury.

## Materials and methods

### Purification of α2-M

The purification of α2-M was performed according to our previous report (Huangfu et al. 2016a). Briefly, Cohn Fraction IV was firstly diluted 10 times using water. Then, the Cohn Fraction IV solution was centrifuged (8000 *g*, 20 min), and the precipitate was removed. (NH_4_)_2_SO_4_ 50% was added to the solution to achieve 50% saturation. Precipitate containing α2-M was obtained after centrifugation (8000 *g*, 15 min). The precipitate was re-suspended using phosphate buffered saline (PBS), and filtered with filter (0.22 μm, Millipore, Molsheim, France). Then, the crude α2-M solution was further separated through immobilized metal affinity chromatography as described (Huangfu et al. 2016a).

### Sodium dodecyl sulphate polyacrylamide gel electrophoresis (SDS-PAGE) and 2D electrophoresis

Protein concentration was detected with BCA method. Different amounts of α2-M and α2-M standard (Purity ≧95%, Sigma, St. Louis, MO) were solubilized with buffer (pH 6.8, 50% glycerol in 250 mmol/L Tris buffer, 100 mg/mL SDS, 5 mg/mL bromophenol blue). Then, SDS-PAGE was conducted using precast 10% Bis–Tris gels on a Mini-Protein Tetra System (Bio-Rad, Hercules, CA). Finally, the proteins were stained using CoomassieBlueG-250 and capture using ImageQuant 350 Digital Imaging System (GE Healthcare, Danderyd, Sweden).

The protein in the Cohn Fraction IV was measured. Immobilized pH gradient strips (18 cm, pH 3–10) were used to separate protein sample. Then, isoelectric focussing electrophoresis was performed after rehydration (15 h). After isoelectric focussing electrophoresis, the buffer (bromophenol blue, 18 mmol/L DTT, 40 mmol/L Tris–HCl, 1.5% SDS, 25% glycerol and 4 mol/L urea) was used for equilibration. The gels were silver stained and analysed using Imagemaster 2D Pultinum version 5.0 (Boston, US); ProteinLynx Global Server version 2.5 (PLGS 2, Milford, US).

### LC-ESI-MS/MS analysis

The LC-ESI-MS/MS analysis was conducted as described previously (Huangfu et al. 2016a). Briefly, the target protein band was cut into 1 mm^3^ pieces, and digested using trypsin. The trypsinized protein was transferred to the precolumn. Then, the peptides were separated using linear gradient of 10–40% buffer A (0.1% formic acid in acetonitrile). A mass spectrometer (SYNAPT G2-S, Waters, Milford, MA) was used for peptides analysis. MS/MS and mass LC-MS data were collected in high-definition DDA mode. ProteinLynx Global Server version 2.5 (PLGS 2.5) was used to process data. NCBI protein database (June, 2021) was used for analysis.

### Detection of cell proliferation through the CCK8 method

Human skin fibroblasts (ATCC, Manassas, VA) were used in this research for *in vitro* study. 1 × 10^4^ cells were plated into a 96-well plate. After culture for 24 h, DMEM medium was removed, and cells were treated with irradiation (10 Gy). DMEM was added, and cells were treated with α2-M for 24 h. Then, cells were incubated with CCK-8 regent (Nanjing Jiancheng, Nanjing, China) for 4 h, and OD at 490 nm was detected.

### Measurement of cell migration through wound healing

Of 2 × 10^5^ cells (Human skin fibroblasts) were plated into a 6-well plate. When cells grew to 70% confluence, DMEM medium was removed, and cells were treated with irradiation (10 Gy). Then, 1 mL DMEM medium and α2-M were added and, 200 μL pipette was used to make a scratching line in the middle of well. After culture at 37 °C and 5% CO_2_ for 24 and 48 h, images were captured.

### Assessment of cell invasion through transwell method

Cells (2 × 10^4^) (Human skin fibroblasts) were seeded into the top chamber. After culture for 24 h, DMEM medium was removed, and cells were treated with irradiation (10 Gy). α2-M or normal saline was added to the top chamber used to treat cells, and 1.5 mL Dulbecco’s Minimal Essential Medium (DMEM) with 15% FBS was added to the lower chamber. After 48 h, polyformaldehyde (4%) was used to fix cells. After washing with PBS, cells were stained with Giemsa. Finally, cells were observed with an inverted microscope.

### Detection of cell ROS level

Human skin fibroblasts 1 × 10^4^ cells were plated into a 96-well plate. After culture for 24 h, DMEM medium was removed, and cells were treated with irradiation (10 Gy). Then, cells were treated with α2-M for 24 h. Fluorescent DCF dye (DCFH-DA; 2 μM, Invitrogen, Carlsbad, CA) was added, and incubated for 20 min in the dark. After washing with PBS twice, ROS levels were observed using a fluorescence microscopy.

### Measurement of MDA, SOD and CAT

The levels of MDA, SOD and CAT were measured using related ELISA kits according to the instructions. The ELISA kits used in this study were as follows: MDA (#A003-1-1, Nanjing Jiancheng, China), SOD (#A001-1-1, Nanjing Jiancheng, China), and CAT (#A007-1-1, Nanjing Jiancheng, China).

### Western blotting

Cellular protein was extracted with a cell lysate. Samples were separated using 12% SDS-PAGE. After transferring to nitrocellulose membrane (Millipore, Burlington, MA), the membrane was blocked with TBST containing 5% skim milk powder for 2 h. After washing with PBS for 3 times, membrane was incubated with primary antibodies (1:1000) overnight at 4 °C. After washing with PBS for 3 times, membrane was incubated with secondary antibodies (1:2000) for 4 h at 25 °C. Protein bands were analysed through Image J software. The antibodies used in this study were listed as follows: rabbit monoclonal to H2A histone family member X (H2AX, Abcam, Cambridge, UK; #ab229914), rabbit polyclonal to transient receptor potential melastatin 2 (TRPM2, Abcam, #ab11168), rabbit polyclonal to GAPDH (Abcam, #ab9485) and goat anti-rabbit IgG antibody (ab205718).

### Immunofluorescence staining

Cells were first fixed using paraformaldehyde (4%), and then permeated with Triton X-100 (0.06%). After blocking with 3% skim milk, cells were incubated with fluorescein-conjugated anti TRPM2 antibody (#PA1-46466, Invitrogen, Carlsbad, CA) at 4 °C. DAPI was used to stain cell nuclei. Immunofluorescence expression was observed through a fluorescence microscope.

### Measurement of cell apoptosis, mitochondrial membrane potential, calcium levels and cell cycle by flow cytometry

Cells were seeded and cultured as described above. After treatment with irradiation (10 Gy) and α2-M, cells were incubated with Annexin V-FITC and propidium iodide (Nanjing Jiancheng, Nanjing, China), JC-1 dye (3 µM, Beyotime, Nantong, China), and fluo-4AM (Invitrogen, Carlsbad, CA), respectively, for measuring cell apoptosis, mitochondrial membrane potential and calcium level. After incubation at 37 °C and 5% CO_2_ for 20 min, flow cytometry was performed to measure cell apoptosis, mitochondrial membrane potential and calcium level, respectively.

For the detection of cell cycle, cells were fixed with 70% ethanol at 4 °C for 4 h after treatment with irradiation (10 Gy) and α2-M. RNase A (Solarbio, Beijing, China) was added to treat cells, and then propidium iodide (5 µL, Nanjing Jiancheng Bioengineering Institute, Nanjing, China) was added to incubate cells for 20 min. Finally, cell cycle was analysed using a flow cytometer.

### Statistical analysis

Data were presented as mean ± standard deviation, and analysed using SPSS version 22.0 (SPSS Inc., Chicago, IL). Statistical significance was determined using ANOVA. *p* < 0.05 indicates statistically significant.

## Results

### Purification and identification of α2-M

**Figure 1.**
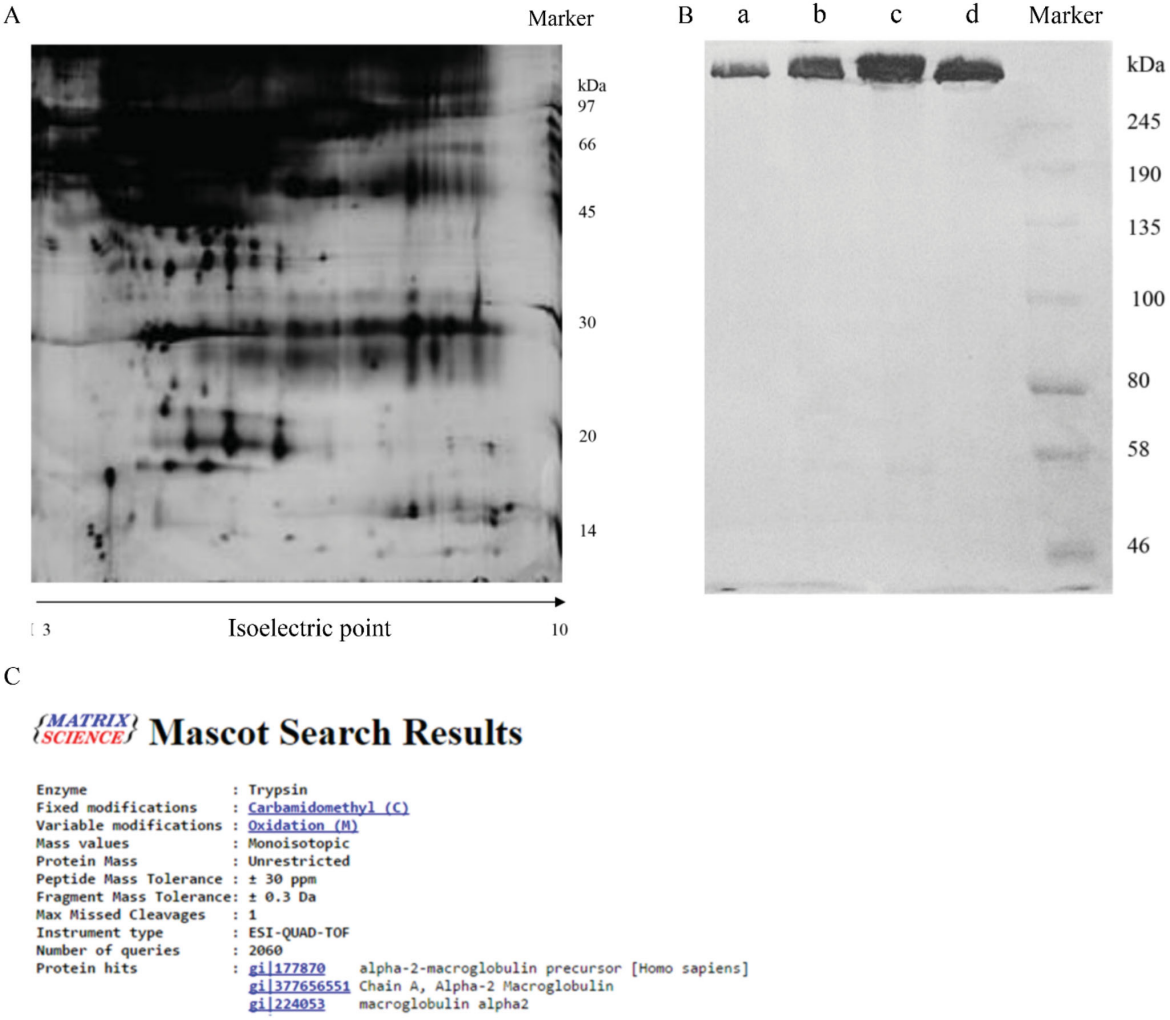
Purification and identification of α2-M. A: Identification of Cohn fraction IV through two-dimensional electrophoresis; B: Purity identification of final product through SDS-PAGE (a: 5 µg final product, b: 10 µg final product, c: 15 µg final product, d: 15 µg α2-M standard); C: Identification of final product through LC-ESI-MS/MS analysis.

The α2-M used in this study was purified from Cohn fraction IV, which contains more than 200 types of plasma proteins. The components of Cohn fraction IV were investigated through 2D electrophoresis. Many different kinds of proteins were observed through 2D electrophoresis (Figure 1(A)). After purification processes, similar purity between the final product we isolated and α2-M standard with more than 95% purity was observed (Figure 1(B)). In addition, qualitative analysis was performed to identify the final product. The top 3 proteins were α2-M-related proteins (Figure 1(C)) through LC-ESI-MS/MS analysis (Huangfu et al. 2016a) indicating that high purity of α2-M was successfully prepared.

### α2-M markedly promoted the cell proliferation and migration on the dose dependent manner

**Figure 2.**
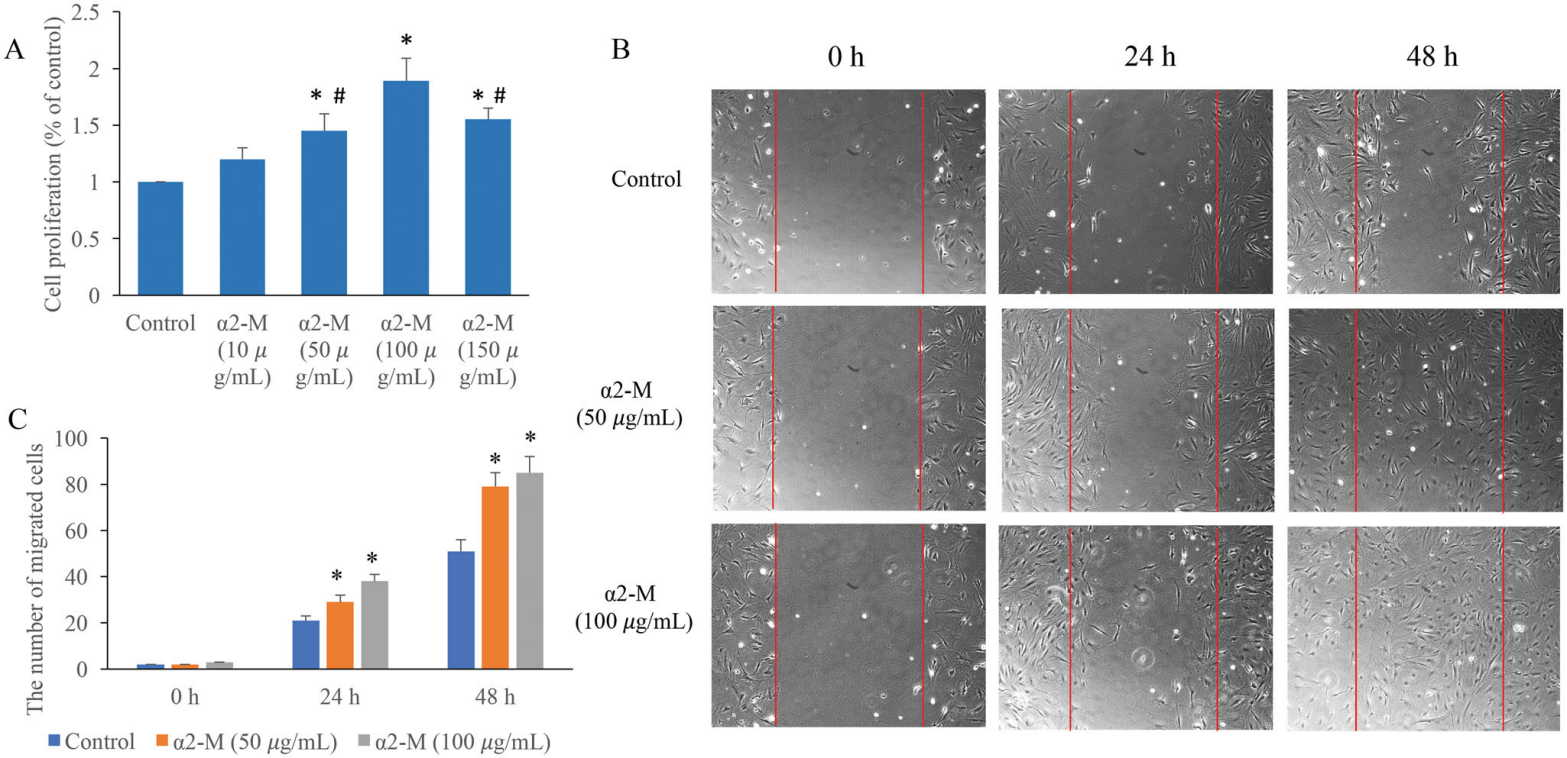
α2-M markedly promoted the cell proliferation and migration. A: Cell proliferation was measured after α2-M treatment; B: Cell migration was measured after α2-M treatment; C: α2-M markedly promoted the cell migration on the dose-dependent manner. *p < 0.05 compared with group control. ^#^*p* < 0.05 compared with group α2-M (100 µg/mL).

Fibroblasts are closely related with skin repair after irradiation damage, so fibroblasts were used in this study to investigate the role of α2-M in the improvement of irradiation damage. We found that medium (50 µg/mL) and high concentration (100 µg/mL) of α2-M could remarkably promote the ability of fibroblasts proliferation (Figure 2(A)) and migration (Figure 2(B,C)). These results indicate that α2-M might be involved in the regulation of fibroblasts viability. In addition, the proliferation ability of cells in the group α2-M (150 µg/mL) was significant lower compared with group α2-M (100 µg/mL). Therefore, the dose of 100 µg/mL was chosen in the following experiments.

### α2-M significantly reversed the influence of irradiation on cell viability

**Figure 3.**
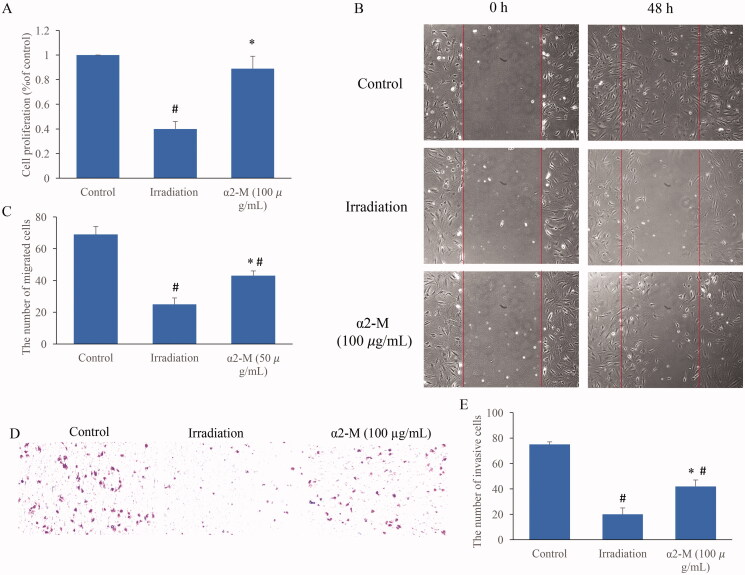
α2-M significantly reversed the influence of irradiation on cell viability. A: Cell proliferation was measured after irradiation and α2-M treatment; B: Cell migration was measured after irradiation and α2-M treatment; C: α2-M markedly promoted the cell migration after irradiation; D: Cell invasion was measured after irradiation and α2-M treatment; E: α2-M markedly promoted the cell invasion after irradiation. *p < 0.05 compared with group irradiation. ^#^*p* < 0.05 compared with group control.

To further unfold the regulation of α2-M in irradiation-induced injury, irradiation (10 Gy) induced cell damage model was established. After irradiation treatment, the cell proliferation was markedly suppressed (Figure 3(A)). However, treatment with α2-M (100 µg/mL) significantly reversed the influence of irradiation and increased the ability of cell proliferation. In addition, we found that α2-M (100 µg/mL) remarkably promoted the cell migration and invasion ability after irradiation (Figure 3(B–E)).

### α2-M markedly reversed the affection of irradiation on cell apoptosis and cycle

**Figure 4.**
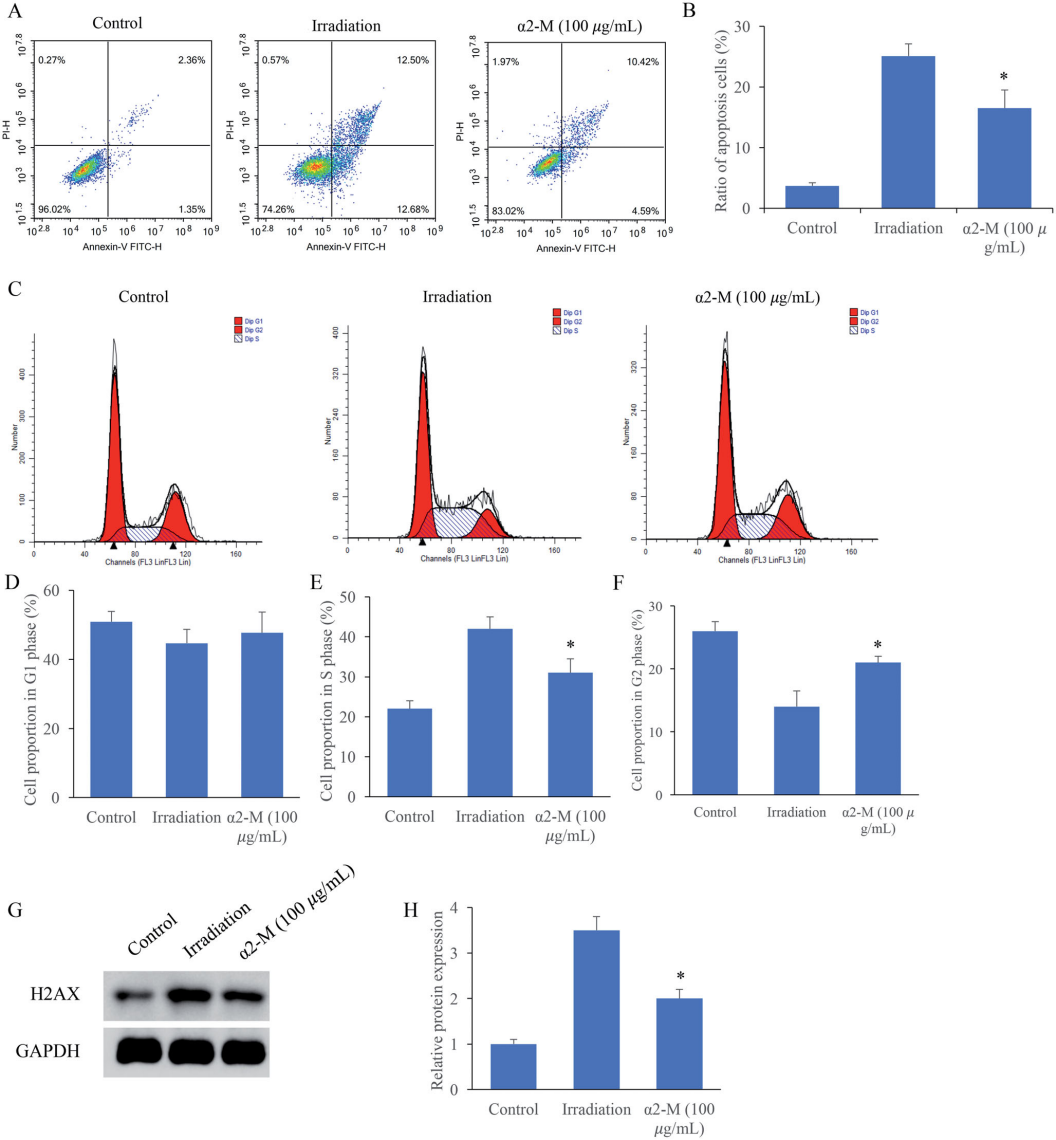
α2-M markedly reversed the affection of irradiation on cell apoptosis and cycle. A: Cell apoptosis was measured after irradiation and α2-M treatment; B: α2-M significantly suppressed the cell apoptosis after irradiation; C: Cell cycle was measured after irradiation and α2-M treatment; D: Cell ratio in the G1 phase was measured; E: Cell ratio in the S phase was detected; F: Cell ratio in the G2 phase was measured; G: H2AX protein expression was measured *via* western blotting; H: α2-M significantly suppressed H2AX expression after irradiation. *p < 0.05 compared with group irradiation. ^#^*p* < 0.05 compared with group control.

The cell apoptosis and cell cycle were after irradiation and α2-M treatment were also detected. Irradiation markedly induced cell apoptosis, but the trend was suppressed by α2-M (Figure 4(A,B)). Meanwhile, we found that irradiation significantly increased the cell proportion in S phase, but decreased the cell ratio in the G1 and G2 phase (Figure 4(C–F)). However, α2-M markedly reversed the influence of irradiation on cell cycle. In addition, increase of H2AX expression induced by irradiation could inhibited by α2-M (Figure 4(G,H)).

### α2-M markedly suppressed the ROS levels of fibroblasts treated by irradiation

**Figure 5.**
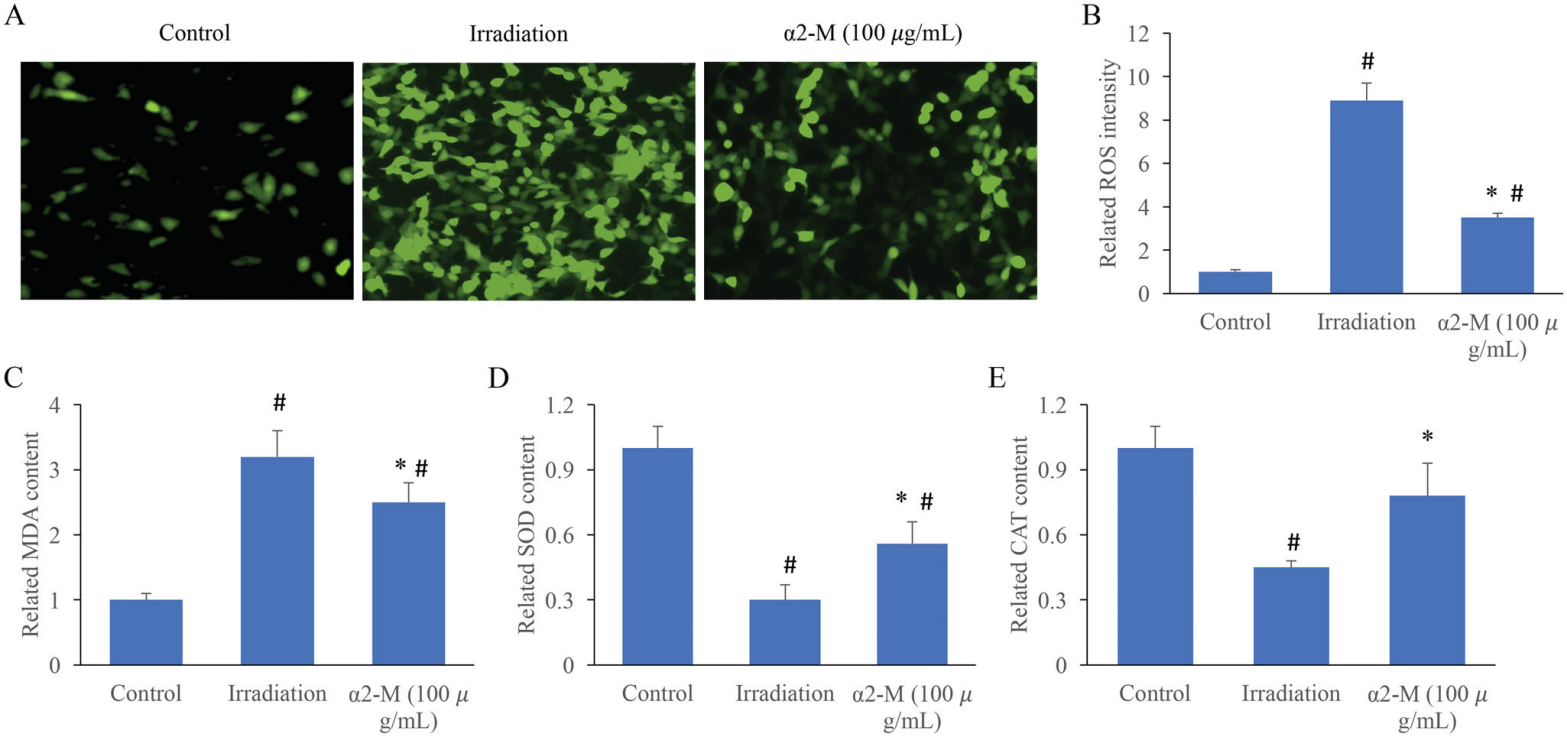
α2-M markedly suppressed the ROS levels of fibroblasts treated by irradiation. A: ROS levels in the fibroblasts were measured; B: ROS intensity in the fibroblasts were analysed; C: MDA contents in the fibroblasts were measured; D: SOD levels in the fibroblasts were measured; E: CAT concentration in the fibroblasts were measured. *p < 0.05 compared with group irradiation. ^#^*p* < 0.05 compared with group control.

The redox balance in the cells was also investigated. Of 10 Gy irradiation markedly increased the ROS levels, but α2-M remarkably suppressed it (Figure 5(A,B)). Meanwhile, increase of MDA, and decrease of SOD and CAT, were induced by irradiation. However, α2-M significantly increased CAT and SOD, but suppress MDA compared with group irradiation (Figure 5(C–E)).

### α2-M significantly improved the mitochondrial damage induced by irradiation

**Figure 6.**
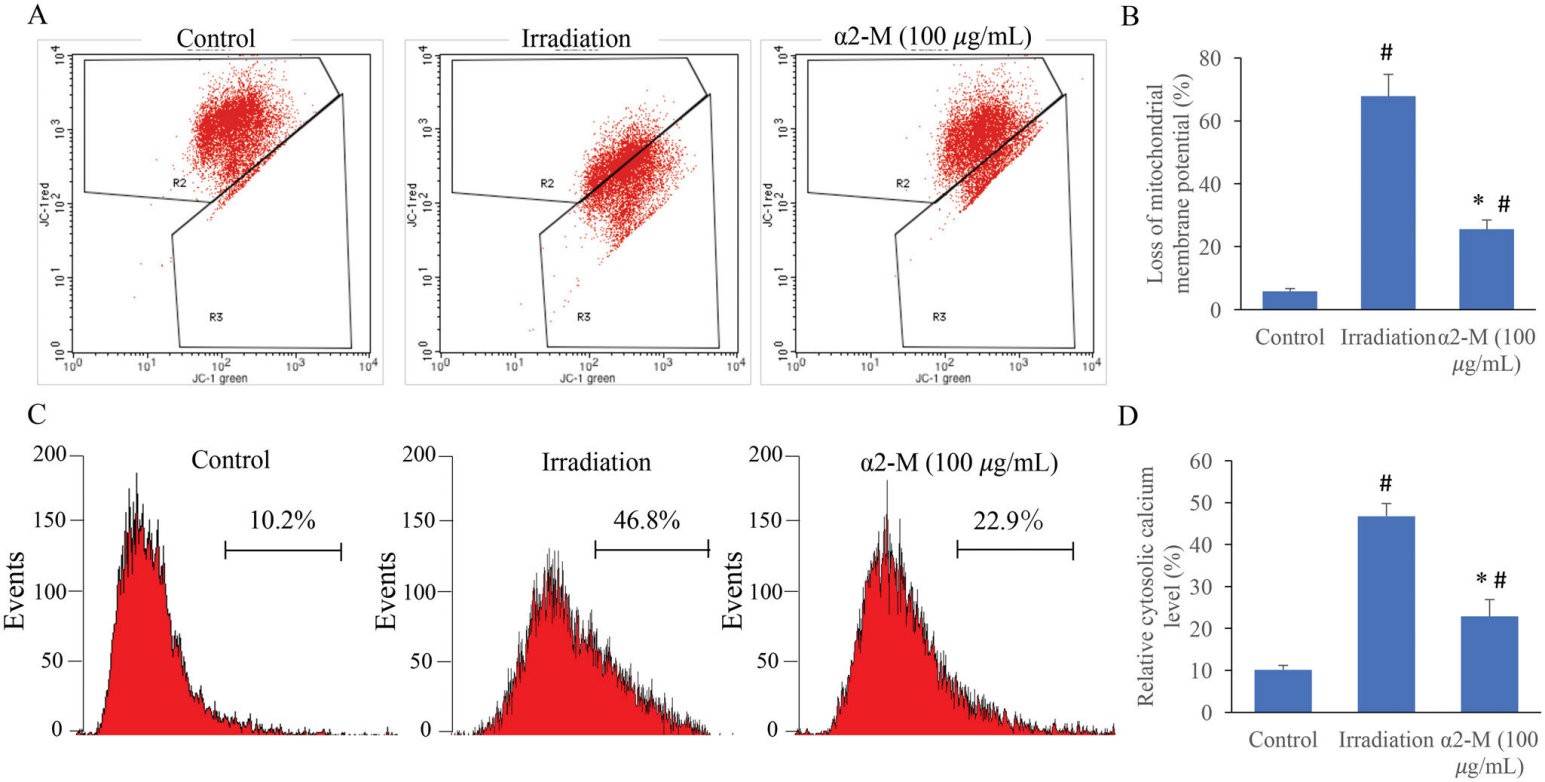
α2-M significantly reduced the mitochondrial damage induced by irradiation. A: Mitochondrial membrane potential levels were measured through flow cytometry; B: Mitochondrial membrane potential levels were analysed quantitatively; C: Calcium contents were measured through flow cytometry; D: Calcium contents were analysed quantitatively. *p < 0.05 compared with group irradiation. ^#^*p* < 0.05 compared with group control.

The levels of mitochondrial membrane potential (MMP) and calcium are closely linked with mitochondrial function. We found that irradiation could lead to remarkable increase of MMP loss and calcium, but the influences were significantly weakened by α2-M treatment (Figure 6(A–D)).

### The level of TRPM2 was markedly inhibited by α2-M after irradiation

**Figure 7.**
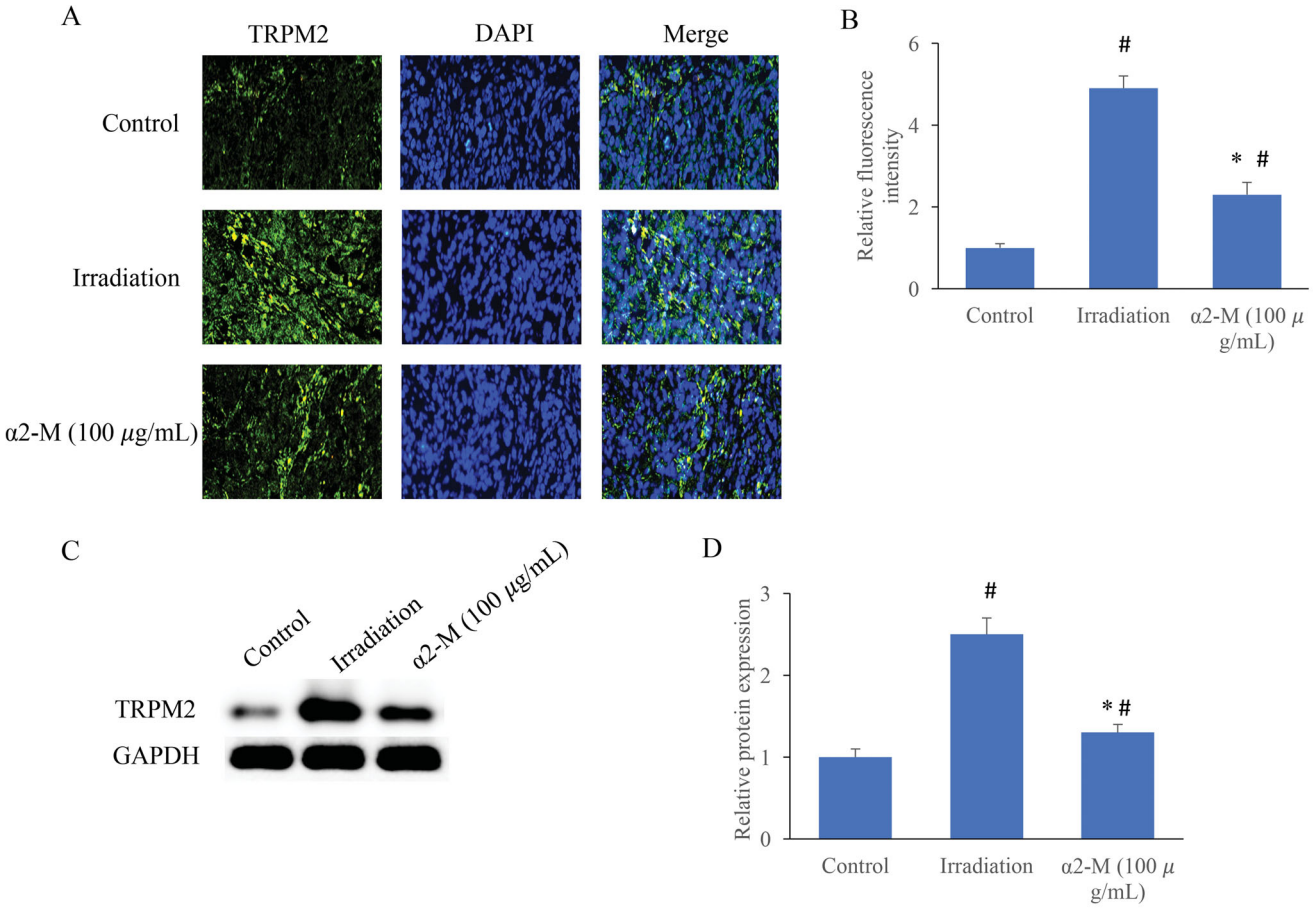
The level of TRPM2 was markedly inhibited by α2-M after irradiation. A: The TRPM2 expression was measured through immunofluorescence staining; B: The TRPM2 expression was analysed quantitatively; C: The TRPM2 expression was measured through western blotting; D: The TRPM2 expression was analysed quantitatively. *p < 0.05 compared with group irradiation. ^#^*p* < 0.05 compared with group control.

In addition, the TRPM2 expression in the cells was investigated. Significant higher expression of TRPM2 was observed in the group irradiation. However, α2-M significantly inhibited TRPM2 compared with group irradiation (Figure 7(A–D)).

## Discussion

α2-M plays an important role in several physiological and pathological processes. For example, as a broad-spectrum protease inhibitor, α2-M could reduce excessive endogenous and exogenous proteases (Huangfu et al. 2017). Meanwhile, the anti-radiation and antitumor effects of α2-M have been confirmed by some reports. α2-M is involved in blood coagulation balance system and has the function of inhibiting oxygen free radicals. In addition, after interacting with cytokines, α2-M could affect their biological activity (Rehman et al. 2013).

Radiation therapy has been widely used in the field of tumour treatment. However, during the process of radiation therapy, normal tissues and cells along with cancers cells are also eliminated by irradiation. Therefore, safe and effective agents with anti-radiation function need to be explored. Several studies have confirmed the effect of α2-M on radiation protection. Remarkable lower concentration of α2-M was observed in the serum of jaws osteoradionecrosis patients (Liu et al. 2018). Significant increase of rat survival rate from 50 to 100% was achieved by α2-M treatment intraperitoneally after radiation (Bogojevic et al. 2011). Amifostine is a type of anti-radiation agent, and amifostine could markedly increase the α2-M level in the rat after irradiation. Therefore, α2-M might act an important role in the amifostine-induced anti-irradiation effect (Mirjana et al. 2010). The DNA injury caused by irradiation is viewed to be the trigger of subsequent cell dysfunction events. Proliferating cell nuclear antigen (PCNA) plays a vital role during DNA repair process (Seelinger and Otterlei 2020), and α2-M could significantly promote the expression of PCNA and suppress DNA damage in the irradiation-treated rats (Bogojevic et al. 2011).

Irradiation could lead to the production of ROS in cells and tissues, and ROS is the reflection of oxidative stress (Hernandez et al. 2019; Yuan et al. 2019). In addition, ROS could change the levels of cholesterol and fatty acids on the cell membrane, and further lead to cell apoptosis (Wang, Bian, et al. 2017). Some enzymes, such as SOD, CAT and MDA could maintain the redox system, and regulate the levels of ROS. Especially, SOD could catalyse superoxide anion into hydrogen peroxide and oxygen leading to the inactivation of ROS (Fındıklı et al. 2018). In this study, we demonstrated that α2-M remarkably suppressed the increased ROS levels caused by irradiation (Figure 5(A,B)). Meanwhile, some reductases (CAT and SOD) were increased, but MDA was inhibited by α2-M (Figure 5(C–E)). The inhibition of cell apoptosis by α2-M might be explained by the suppression of ROS intensity.

Mitochondria are closely related with cell apoptosis, calcium balance, regulation of redox and synthesis of ATP. Dysfunction of mitochondria might cause a series of pathological events. In addition, the mutation of mitochondria DNA may be inherited to next generation (Wang et al. 2019). Irradiation could induce mitochondria damage through promoting mitochondrial fission (Jin et al. 2018). The ROS increase induced by irradiation could result in loss of mitochondrial membrane potential, cytochrome c release and caspase cascade reaction, and further induce cell apoptosis (Sharaf et al. 2017). TRPM2 is an unselective calcium channel, which is sensitive to ROS increase. The oxidative stress caused by ROS increase could activate TRPM2 and increase calcium content leading to cell damage (Wang, Huang, et al. 2017; Alves-Lopes et al. 2020). The inhibition of mitochondrial membrane potential loss and TRPM2 expression after irradiation might account for the anti-irradiation effect of α2-M.

The damage caused by irradiation is believed to be closely related to the production of some inflammatory factors including TNF-α, IL-6 and IL-1 (Sultan et al. 2013; Wang et al. 2020). α2-M is proven to be a carrier for some cytokines. For example, α2-M could bind to IL-6 (Nancey et al. 2008), IL-1β (Athippozhy et al. 2011) and TNF-α (Jeng et al. 2011), and further influence their function. Therefore, if α2-M exerts its radiation-protective effect through binding some cytokines remains unclear, which should be an interesting research direction. The lack of animal experiment validation is a limitation of this research.

## Conclusions

**Figure 8.**
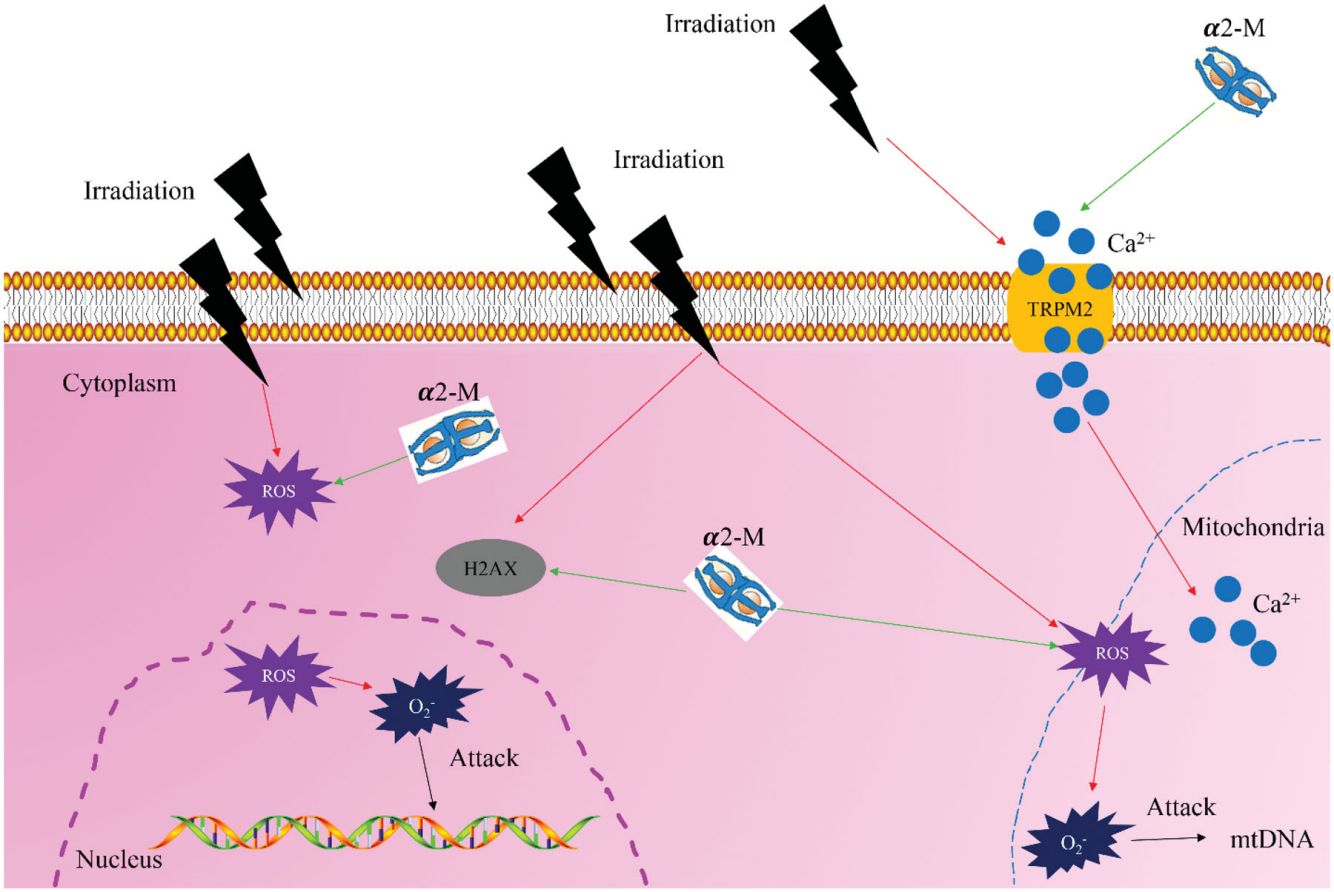
Schematic hypothesis of improvement of irradiation-induced damage by α2-M (red arrow indicates promotion effect and green arrow suggests suppression effect).

This study demonstrated that α2-M remarkably increased cell migration, invasion and proliferation, but inhibits cell apoptosis after irradiation treatment. Meanwhile, the increased ROS and oxidative stress level induced by irradiation could be suppressed by α2-M. In addition, α2-M could decrease the levels of MMP, calcium and TRPM2 to alleviate mitochondrial damage caused by irradiation (Figure 8). Therefore, α2-M might be a promising anti-irradiation agent for the treatment of tumour therapy.

## Data Availability

The data supporting this study can be requested from corresponding author.
